# Beyond Screen Time: Smartphone Dependency, Sleep Disruption, and Emotional Regulation Among Young Adults—A Cross-Sectional Web-Based Survey

**DOI:** 10.3390/healthcare14182982

**Published:** 2026-09-12

**Authors:** Sudharani B. Banappagoudar, Nader Alnomasy, Shimaa Ramadan Ahmed, Ahmed Ali Hafez

**Affiliations:** 1 Department of Nursing, College of Applied Medical Sciences, King Faisal University, Al Asha 36362, Saudi Arabia; srahmed@kfu.edu.sa; 2 Medical Surgical Nursing Department, College of Nursing, University of Hail, Ha’il 81422, Saudi Arabia; 3 Frances Payne Bolton School of Nursing, Case Western Reserve University, Cleveland, OH 44106, USA; 4 College of Nursing, Northern Border University, Arar 73211, Saudi Arabia; ahmed.hafez@nbu.edu.sa

**Keywords:** smartphone use, sleep quality, emotion regulation, young adults, nursing, digital health

## Abstract

**Background:** Smartphones are ubiquitous among young adults today. However, many young adults exhibit characteristics of problematic or dependent phone use. These individuals often have sleep disturbances and difficulties with emotional regulation. Currently, the research on the connection between these three variables is scarce, with particular gaps in studies of nursing professionals in this population. **Objective:** The study aimed to identify the prevalence of problematic phone use among young adults and the relationship between these three variables: problematic phone use, sleep disturbances, and difficulties with emotion regulation. The study will also investigate the potential of sleep disturbances to mediate the relationship between problematic phone use and difficulties with emotion regulation. **Methods:** Data was collected from participants aged 18 years or older through a self-administered, anonymous web survey. The web survey was disseminated via social media and used snowball sampling to collect responses from individuals from various regions. There were no investigators involved in data collection from any particular institution or to reduce researcher bias. A power analysis was performed to determine the ideal sample size; the results from the 167 participants exceeded the ideal sample size requirement. The Smartphone Addiction Scale–Short Version (SAS-SV), a sleep disruption score derived from the Pittsburgh Sleep Quality Index (PSQI), and the Difficulties in Emotion Regulation Scale–Short Form (DERS-SF) were used to collect data. Data were analyzed using descriptive and inferential statistics, including Cronbach’s alpha, Pearson correlations, independent-samples *t* tests, one-way ANOVA, multiple linear regression, and bootstrapped mediation (*n* = 5000 resamples). The CHERRIES guidelines were adhered to throughout the research study. **Results:** The participants were aged a mean of 21.6 years old (SD = 4.6). The majority of the participants (n = 117, 70.1%) identified as female. The internal consistency reliability for the SAS-SV and DERS-SF was high (SAS-SV α = 0.94; DERS-SF α = 0.88). Based upon the sex-specific cutoffs for the SAS-SV, a total of 63 participants (37.7%) screened positive for problematic phone use. There were significant positive correlations between problematic phone use and sleep disturbances (r = 0.41, *p* < 0.001) as well as problematic phone use and difficulties with emotion regulation (r = 0.54, *p* < 0.001). There was a significant positive correlation between sleep disturbances and difficulties with emotion regulation (r = 0.37, *p* < 0.001). The amount of time each individual spent on their smartphone on an average daily basis differed significantly across self-reported daily use bands (F_2,164_ = 6.26, *p* = 0.002), with dependency highest among those reporting more than 5 h of daily use. There were no significant differences in this relationship based upon sex. Multiple linear regression analysis revealed that problematic phone use was a significant predictor of difficulties with emotion regulation (β = 0.47, *p* < 0.001); once the effects of problematic phone use were accounted for, sleep disturbances also significantly predicted difficulties with emotion regulation (β = 0.18, *p* = 0.01). In a complete-case analysis excluding 12 participants with uninterpretable sleep-duration data (*n* = 155), the bootstrapped indirect effect of sleep disturbances on the relationship between problematic phone use and difficulties with emotion regulation was statistically significant (ab = 0.081, 95% CI = 0.015 to 0.174, adjusting for age and sex); an earlier analysis that had scored these 12 cases as zero rather than excluding them found a smaller, non-significant indirect effect (ab = 0.060, 95% CI = −0.014 to 0.140). **Conclusions:** Problematic phone use is common among young adults today. The use of problematic phone behavior is positively associated with sleep disturbances and difficulties with emotion regulation. In a corrected complete-case analysis, sleep disturbances significantly mediated part of this relationship, though this cross-sectional association should not be interpreted as demonstrating a causal mechanism, and it should be weighed against a smaller, non-significant estimate obtained before this missing-data correction. These findings suggest that both problematic phone use and, cautiously, sleep health may be relevant targets for nursing-led digital-wellness interventions, though the effectiveness of such interventions would need to be established in prospective studies before implementation.

## 1. Introduction

### 1.1. Background

Smartphones are ubiquitous among young adults of today. In the past decade, smartphone use among adolescents and young adults has increased exponentially and has become one of the essential devices of the young adults in their life [1,2]. Young adults are the most intensive users of smartphones. At this life stage, the adolescent brain is developing and is most sensitive to stimuli in the environment and to the reward systems in the brain [2]. The smartphone’s features that make it essential for young adults also make it addictive and create a problem of problematic smartphone use [3,4,5].

The dependency of individuals on their smartphones is distinct from the amount of time that they spend on their smartphones. Problematic phone use is defined as losing control of the time spent on their smartphones, the salience of their smartphones in their life, the tolerance they develop to the amount of time spent on their smartphones, the development of symptoms of withdrawal when separated from their smartphones, and the continued use of their smartphones despite the negative impacts on their life and mental health [3,4,5]. The Smartphone Addiction Scale–Short Version (SAS-SV) was developed to measure the level of dependency of individuals on their smartphones, not the amount of time that they spend on their mobile devices [6].

The dependency of young adults on their smartphones has two main impacts upon their lives: their sleep and their ability to regulate their emotions [7]. Sleep disturbances are often reported among young adults with problematic phone use. The time that young adults spend before sleeping on their mobile devices is often reported to impact their sleep patterns [8,9,10]. One study has indicated that the impact of problematic phone use upon the sleep patterns of young adults is often outsized compared to other populations that may be less dependent upon their mobile devices [10]. The effects of sleep deprivation on young adults can include the development of various sleep disorders and difficulties in regulating sleep patterns [9]. Additionally, problematic phone use is linked to difficulties with emotion regulation. Young adults with a history of problematic phone use often struggle to regulate their emotions due to a reliance on their smartphones to cope with stress and regulate their emotions in a negative manner [11,12]. Studies have linked emotional dysregulation to the development of mental health issues, such as anxiety and depression [13].

A hypothesis has been formed linking the impacts of problematic phone use upon sleep and emotion regulation. It is hypothesized that problematic phone use will impact the sleep patterns of young adults. Additionally, these sleep disturbances will impact the ability of young adults to regulate their emotions, a pathway with plausible neurobiological grounding: experimentally induced sleep loss weakens prefrontal cortex regulation of amygdala reactivity, impairing top-down emotional control [14]. This proposed mechanism is consistent with meta-analytic evidence of a moderate, well-replicated association between emotion-dysregulation and problematic smartphone use more broadly (pooled r ≈ 0.42) [15]. It has been hypothesized that the relationship between problematic phone use and difficulties with emotion regulation will be mediated by the impact of problematic phone use upon the sleep of young adults [16,17].

The current study on this issue is motivated by two specific gaps in the existing literature. First, most of the literature on this topic has focused upon the amount of time that individuals use their smartphones rather than their level of dependency upon their mobile devices. Second, there is a scarcity of nursing professionals working with young adults who have been studied in relation to the connection between problematic phone use, sleep disturbances, and difficulties with emotion regulation [18]. Therefore, research is needed on the connection between these three variables.

### 1.2. Objectives and Hypotheses

The primary objectives of this study are to: (1) estimate the prevalence of problematic smartphone use among young adults; (2) to examine the sleep disturbances and difficulties with emotion regulation of young adults with problematic smartphone use; (3) to examine the correlation between problematic phone use and sleep disturbances as well as the correlation between problematic phone use and difficulties with emotion regulation; (4) to examine the correlation between sleep disturbances and difficulties with emotion regulation; (5) to identify the predictors of difficulties with emotion regulation of young adults; and (6) to determine if the sleep disturbances of young adults mediated their problematic phone use and their difficulties with emotion regulation. The hypotheses formed in relation to these objectives are the following: problematic phone use is associated with sleep disturbances (H1); problematic phone use is associated with difficulties with emotion regulation (H2); sleep disturbances are associated with difficulties with emotion regulation (H3); and sleep disturbances mediate the association between problematic phone use and difficulties with emotion regulation (H4).

## 2. Methods

### 2.1. Study Design

This study utilized a cross-sectional, descriptive-analytical study design in which a self-administered, anonymous web survey was utilized to collect data from young adults. There was no intervention in the study, as the survey was observational. The reporting of the data within the study adhered to the CHERRIES guidelines for online surveys and the STROBE statement guidelines for observational studies with cross-sectional designs [19]. The CHERRIES checklist that was utilized in the data collection and analysis of this study is located as Multimedia Supplementary Materials.

### 2.2. Recruitment, Setting, and Researcher Independence from Data Collection

The data for this study were not collected from individuals from a specific institution. Instead, the self-administered, anonymous survey was disseminated via social media networks where individuals from various regions could participate. There was no geographic restriction on eligibility or on access to the survey: the questionnaire was hosted online in English and its link was openly available, so it could be accessed and completed by any eligible adult, in any country, who received it through social-media dissemination or subsequent snowball sharing. The study therefore had no predefined national or regional scope; the resulting geographic composition of the sample is reported in Section 2.3. This use of snowball sampling and dissemination through social media eliminated the need for investigators to interact with any of the participants in this study. This independence of investigators from the collection of the data reduced the potential for interviewer-related bias toward specific participants and may have reduced social-desirability bias on the part of those participants in completing the survey [3,4,5], though the anonymous, self-selecting recruitment strategy introduces its own potential for self-selection and coverage bias, discussed further in the Limitations. All survey items were closed-ended and published in the same order for all participants. Data collected did not include any information about the identity of any participant, their name, their email address, the IP address from which they submitted their responses, or any information about those individuals that could be traced back to the investigators themselves. These measures were implemented to mitigate the biases that are often associated with the interaction of investigators with their participants, particularly those with institutional affiliation with their participants [19]. The limitations of this study regarding the presence of self-selection of those individuals who participated in the study is considered in the study limitations section below.

### 2.3. Participants and Eligibility

Participants in this study had to be 18 years of age or older and have a smartphone. Exclusion criteria included the presence of a sleep disorder or other mental health issue, or the taking of medications that could impact the sleep patterns or emotional regulation of those individuals. A survey was implemented to identify potential participants that met these criteria, and those that indicated that they did not meet these criteria were excluded from participation in the study. A total of 172 responses were collected from individuals for this study; however, three individuals indicated that they did not wish to participate in the study, and another two indicated that their responses were not eligible for inclusion in the study. Finally, data was removed from individuals for whom some of the required data items for analysis were missing, leaving a total of 167 participants in the study. This study’s sample comprised adults aged 18–49 years, predominantly young adults (mean age 21.6 years; only 8 of 167 participants were older than 30). Participants self-reported their country of residence via an open-text field; of 167 respondents, 83 (49.7%) reported Saudi Arabia, 11 (6.6%) Egypt, and 6 (3.6%) India, while 67 (40.1%) left this field blank or gave an uninterpretable response, so the geographic composition of the remaining sample cannot be fully characterized. All eligibility and exclusion criteria were assessed via closed-ended, self-reported items administered before Section B; no clinical or diagnostic verification was performed, consistent with the study’s anonymous, non-institutional design. “Other mental health issue” was participant-defined and not further specified.

### 2.4. Sample Size and Statistical Power

Sample size was determined prior to the study to ensure that the desired statistical power was achieved to detect the expected effects. Based on Cohen’s conventions for effect size [20], a sample size of 85 participants was required to detect a medium correlation (r = 0.30). Similarly, for the multiple linear regression with four predictors, the same sample size was required for a medium effect size (f^2^ = 0.15). As such, the achieved sample of 167 participants was roughly twice the required sample size for these primary analyses. Furthermore, a power analysis confirmed that the achieved sample size of N = 167 participants would provide 80% power to detect correlations as small as r = 0.22 and was associated with a power of over 0.99 for all three correlations and for the omnibus regression model. While a sample size of 500 was targeted to allow for the analysis of the mediation model, the obtained sample of 167 met the Cohen criterion requirements for the correlational and regression analyses; the power for the mediation model is discussed in the Limitations section. For the prevalence objective specifically, the achieved sample of N = 167 corresponds to a 95% confidence interval margin of error of ±7.4 percentage points around the observed 37.7% screen-positive rate (95% CI: 30.4–45.1%), which readers should weigh alongside the Cohen-based justification above when judging precision for that objective.

### 2.5. Measures and Data Collection Procedure

The instrument comprised four sections presented in a fixed order. Section A collected sociodemographic data: age (open numeric), gender, country of residence, and self-reported average daily smartphone use in three bands (<2 h, 2–5 h, >5 h).

Section B measured smartphone dependency with the 10-item Smartphone Addiction Scale–Short Version (SAS-SV), a validated screening instrument for problematic smartphone use [6]. Item responses were scored on a 1–6 ordinal metric and summed to a 10–60 total, with higher scores indicating greater dependency. Screen-positive dependency was classified using the validated sex-specific cut-offs (≥31 for men, ≥33 for women) [6].

Section C measured sleep disruption with items drawn from the Pittsburgh Sleep Quality Index (PSQI) [21]. Because open-text bedtime and rise-time entries were internally inconsistent and frequently uninterpretable, a five-component PSQI-derived Sleep Disruption Score was computed from the reliably answered components—subjective sleep quality, sleep duration (parsed from reported hours), sleep disturbances, use of sleep medication, and daytime dysfunction—each scored 0–3 and summed to a 0–15 total, with higher scores indicating more disrupted sleep. The sleep-latency and habitual-sleep-efficiency components were omitted because they depend on the unreliable free-text timing fields; this modification is revisited in the Limitations. These five components are measured on different bases (frequency ratings, categorical duration bins, and medication-use frequency), and were not expected to inter-correlate as strongly as a set of items that measure a single construct; the internal consistency of the composite was modest (α = 0.60, *n* = 155), compared to the individual sleep-disturbance item subscale (α = 0.90; *n* = 155). In the primary complete-case analysis excluding the 7.2% of participants with uninterpretable free-text timing entries (*n* = 155), the sleep–emotion-regulation correlation was r = 0.37; in the initial analysis that instead scored these 12 cases as 0 (N = 167), this correlation was r = 0.32, demonstrating the sensitivity of this estimate to the scoring rule (see Section 4.4), and likewise for the sleep regression coefficient (B = 0.86 in the primary *n* = 155 analysis vs. B = 0.64 in the initial N = 167 analysis).

Section D measured emotion-regulation difficulties with the 18-item Difficulties in Emotion Regulation Scale–Short Form (DERS-SF) [22]. Items were scored 1–5; the three Awareness-subscale items were reverse-coded, and items were summed to an 18–90 total, with higher scores indicating greater difficulty. Data were exported from the survey platform and cleaned in Python 3; age was parsed to a numeric value and screened against the eligibility threshold, and scale totals were computed only for respondents who completed at least 80% of the items in a given scale.

### 2.6. Ethical Considerations

This study was conducted in accordance with the Declaration of Helsinki. The study was approved by The Deanship of Scientific Research, Vice Presidency for Graduate Studies and Scientific Research, King Faisal University, Saudi Arabia. (approval no. KFU-REC-2026-AUG–ETHICS4413) and additionally this study was approved by Faculty of Nursing Research Ethics Committee, Zagazig University (approval no.ID/Zu.Nur.RECH#:434).

Participants were provided with information regarding the study’s purpose, confidentiality of their responses, voluntary participation in the study, and the ability to withdraw from the study at any time without penalty. Informed consent was obtained from each participant prior to the commencement of the survey responses.

### 2.7. Statistical Analysis

All analyses were conducted in Python 3 (pandas, NumPy, SciPy), with statistical significance set at *p* < 0.05 (two-tailed). The analytic subheadings below mirror the corresponding subheadings in the Results, one-to-one, so that the derivation of each reported result is explicit. Normality of continuous variables was assessed with the Shapiro–Wilk test prior to parametric analyses; the three composite scores departed modestly from normality (all *p* < 0.01), a common pattern at this sample size that parametric tests are generally robust to, and this is noted as a caveat alongside the regression-residual diagnostics reported in Section 3.5. Because the three primary correlations (H1–H3) involved multiple comparisons, Bonferroni and Benjamini–Hochberg false-discovery-rate corrections were applied; all three remained statistically significant after both corrections (adjusted *p* < 0.001 in all cases), so conclusions are unchanged by the correction.

***Sample characteristics.*** Sociodemographic variables were summarized as frequencies and percentages for categorical variables and as mean (SD) and range for age.

***Descriptive statistics, reliability, and prevalence.*** Composite scores for the SAS-SV, the PSQI-derived Sleep Disruption Score, and the DERS-SF were summarized as mean (SD) and observed range. Internal consistency for each scale was estimated with Cronbach α. The prevalence of screen-positive smartphone dependency was calculated by applying the sex-specific SAS-SV cut-offs and expressed as a percentage of the analytic sample.

***Associations among constructs.*** Pearson product-moment correlations tested the three pairwise associations corresponding to hypotheses H1–H3 (dependency–sleep, sleep–emotion regulation, and dependency–emotion regulation). Assumptions of linearity and approximate normality were inspected graphically.

***Group differences.*** Differences in each composite by sex were tested with independent-samples Welch *t* tests (with Cohen d as the effect size), and differences across the three daily use bands were tested with one-way analysis of variance (ANOVA).

***Predictors of emotion-regulation difficulties.*** A multiple linear regression modeled DERS-SF total score as a function of smartphone dependency, sleep disruption, age, and sex (female vs. male); age and sex were included based on documented associations with these constructs in prior literature [23,24,25]. Unstandardized (B) and standardized (β) coefficients, standard errors, and model fit (R^2^, adjusted R^2^, and the omnibus F test) are reported.

***Mediation analysis.*** The hypothesized mediation (smartphone dependency → sleep disruption → emotion-regulation difficulties; H4) was examined using a cross-sectional indirect-effect analysis with the product-of-coefficients approach; since all three constructs were measured at the same time, the results should be interpreted as an association-level test and temporal or causal ordering of constructs should not be inferred. Age and sex were included as covariates based on prior evidence of their association with each construct: age and gender affect DERS-SF norms [23], gender differences are well-documented in sleep quality [24], and age is an independent predictor of problematic smartphone use, with gender effects reported inconsistently across studies [25]. Age and sex (female vs. male) were included as covariates in all mediation-model paths (a, b, and c/c′). A path (dependency → sleep), b path (sleep → emotion regulation, adjusting for dependency), total effect (c), and direct effect (c′) were estimated by ordinary least squares, and the indirect effect (ab) was evaluated with a 95% bias-corrected bootstrap confidence interval based on 5000 resamples; an interval excluding zero indicates a statistically significant indirect effect. For the multiple linear regression model (Section 3.5) variance inflation factors, Durbin–Watson statistic, Cook’s distance and Jarque–Bera test of residual normality were used for regression diagnostics.

## 3. Results

### 3.1. Sample Characteristics

A total of 167 adults aged 18–49 years, predominantly young adults, participated in the study (average age of 21.6 years with a standard deviation of 4.6; age range was from 18 to 49 years). Most of the participants identified themselves as female (n  =  117; 70.1%), while only 49 identified as male (29.3%) and one identified themselves as another sex. Nearly half of the participants reported that they used their smartphones for more than 5 h each day (Table 1). Within the corrected n = 155 sample, restricting to participants ≤30 years (n = 147, 8 were older) yielded results similar to the full n = 155 sample: r = 0.411 vs. 0.409 for dependency–sleep; r = 0.534 vs. 0.542 for dependency–ER; and β = 0.473 vs. 0.471 for dependency. The screen-positive rate was comparable (39.5% vs. 38.7%).

### 3.2. Descriptive Statistics, Reliability, and Prevalence

The internal consistency was good-to-excellent for the SAS-SV (α = 0.94) and DERS-SF (α = 0.88), but only modest for the five-component sleep-disruption composite (α = 0.60), which is markedly lower than the 9-item disturbance-subscale α (0.90) reported elsewhere and reflects the composite’s structurally heterogeneous items (Table 2, note a). Of this sample, 63 out of 167 (37.7%) screened positive for smartphone dependency using the sex-specific screening positive cut-off scores from the SAS-SV, reflecting the study sample and not a general estimate of the prevalence of smartphone dependency in the general population as a result of this non-probability recruitment strategy. The average number of hours that participants reported that they slept each night was 6.4 h with a standard deviation of 1.9.

### 3.3. Associations Among Constructs (H1–H3)

**Figure 1:**
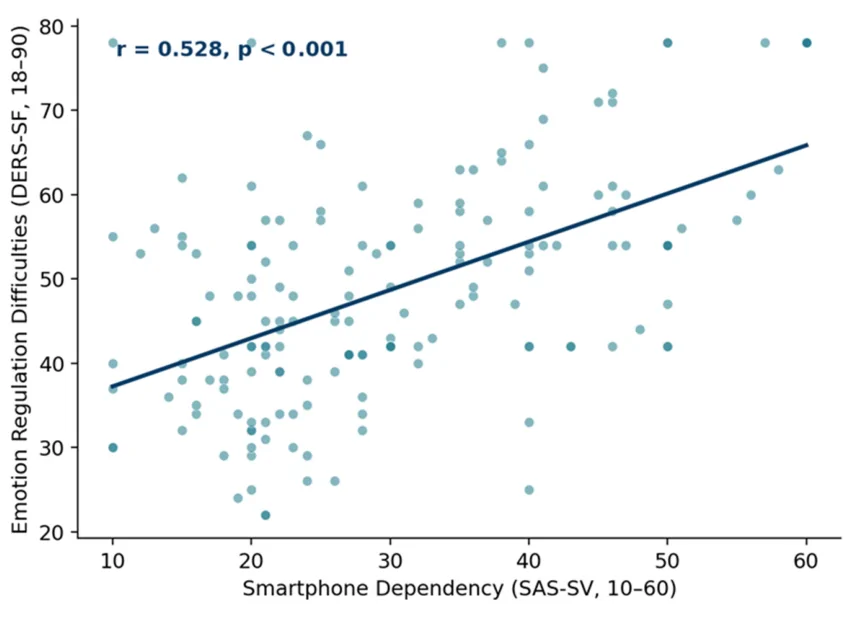
Scatterplot of smartphone dependency (SAS-SV) against emotion-regulation difficulties (DERS-SF) with ordinary-least-squares fit.

All three pairwise correlations between the three constructs were found to be positive and statistically significant for each of the three variables (Table 3, Figure 1). This supports hypotheses 1 through 3 of the present study. Specifically, smartphone dependency was found to be moderately associated with sleep disruption, while smartphone dependency was found to be more strongly associated with emotion-regulation difficulties. Sleep disruption was found to be modestly associated with emotion-regulation difficulties. Pearson correlation assumptions (linearity, approximate bivariate normality) were inspected graphically and considered adequately met; 95% confidence intervals (Fisher z) were: dependency–sleep, 0.41 [0.27, 0.53] (n = 155); dependency–ER, 0.54 [0.42, 0.64] (n = 155); and sleep–ER, 0.37 [0.22, 0.50] (n = 155).

### 3.4. Group Differences

**Figure 2:**
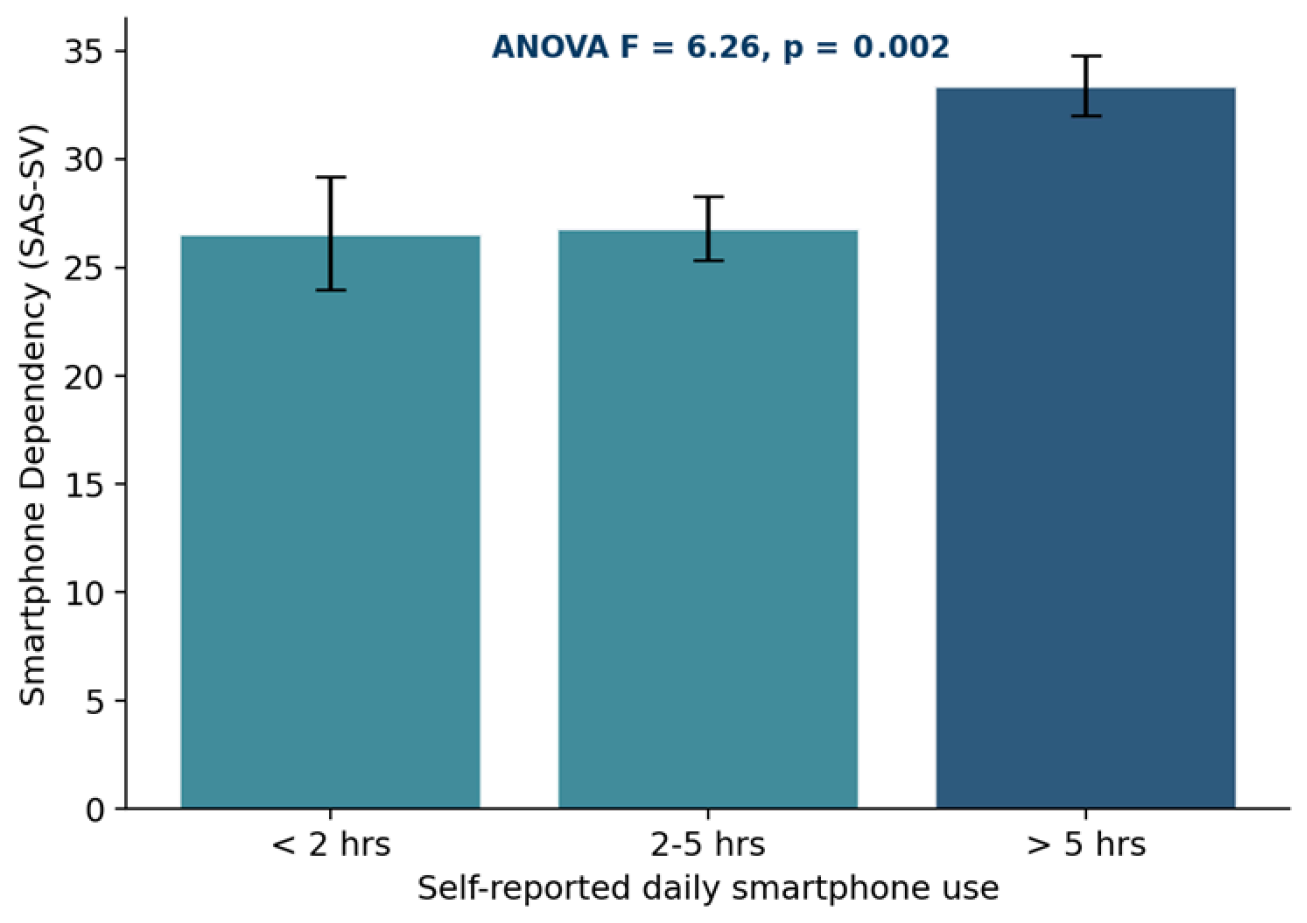
Mean smartphone-dependency (SAS-SV) score by self-reported daily smartphone use. Error bars show ±1 SEM. SAS-SV: Smartphone Addiction Scale–Short Version; SEM: standard error of the mean.

There were no statistically significant differences between males and females on any of the three constructs: smartphone dependency (t  =  −1.50, *p*  =  0.14, Cohen’s d = 0.26), sleep disruption (n = 155; *p*  = 0.41, Cohen’s d = 0.15), and emotion-regulation difficulties (*p*  = 0.84, Cohen’s d = 0.03); all three effect sizes are small by conventional benchmarks. However, significant differences were found in the number of hours that participants reported that they used their smartphones daily with their smartphone dependency score (F_2,164_  =  6.26, *p*  =  0.002, η^2^ = 0.071, ω^2^ = 0.059 a small-to-medium effect), with participants who used their smartphones for more than 5 h per day scoring higher in smartphone dependency than participants who used their smartphones for less than 5 h daily (Figure 2). However, there were no differences in the sleep disruption and emotion-regulation difficulty scores based on the number of hours that participants reported that they used their smartphones daily.

### 3.5. Predictors of Emotion-Regulation Difficulties

The total explained variance of emotion-regulation difficulties was found to be 33% of the participants’ scores (n = 155; R^2^  = 0.33, adjusted R^2^  =  0.31, F_4,150_  =  18.02, *p*  <  0.001). In the corrected (n = 155) analysis, both smartphone dependency and sleep disruption were significant independent predictors of emotion-regulation difficulties. Age and sex were not significant predictors of emotion-regulation difficulties (Table 4). In the corrected (n = 155) model, there was no significant multicollinearity (all variance inflation factors < 1.22), no autocorrelation concern (Durbin–Watson = 1.90), and no individual influential observations (maximum Cook’s distance = 0.08, 5 of 155 observations were outside of the 4/n heuristic threshold, but none were outside of 1.0). Residuals did not show significant non-normality (Jarque–Bera *p* = 0.18), an improvement on the mild non-normality noted in the initial N = 167 model.

### 3.6. Mediation Analysis (H4)

**Figure 3:**
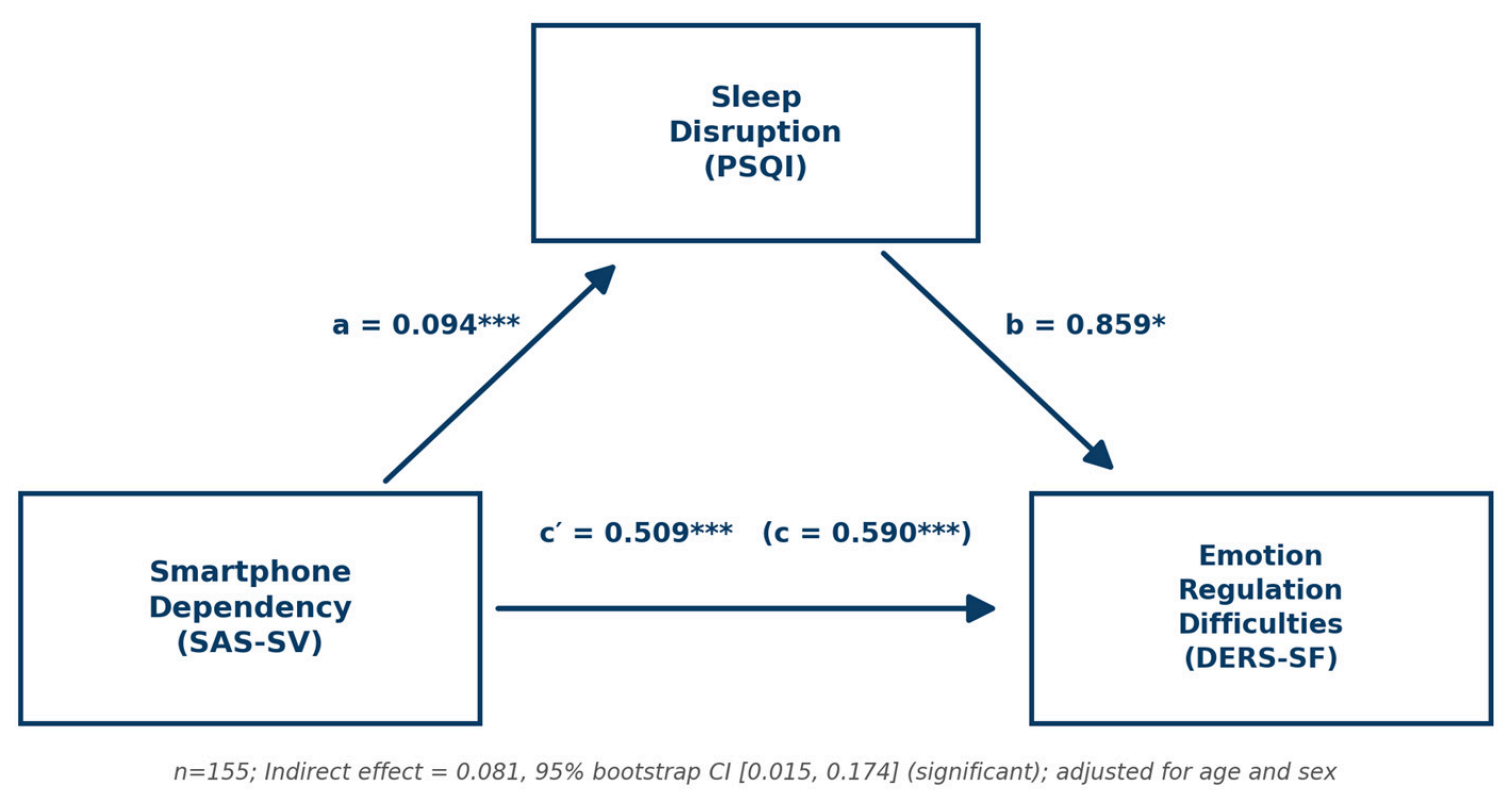
Mediation model of smartphone dependency (SAS-SV), sleep disruption (PSQI-derived), and emotion-regulation difficulties (DERS-SF). Unstandardized coefficients shown; *** *p* < 0.001. n = 155 primary (complete-case) analysis; all paths adjusted for age and sex. * *p* < 0.05. The indirect (mediated) effect was statistically significant.

Following correction of the sleep-duration missing-data handling (Section 2.5), the primary mediation analysis was conducted on the n = 155 complete-case sample. It was found that smartphone dependency significantly predicted sleep disruption (a = 0.094, *p* < 0.001), and the total effect of smartphone dependency on emotion-regulation difficulties was also significant (c = 0.590, *p* < 0.001). When sleep disruption was entered into the equation with smartphone dependency, the direct effect of smartphone dependency on emotion-regulation difficulties remained significant (c′ = 0.509, *p* < 0.001), and the sleep → emotion-regulation difficulties path was also statistically significant (b = 0.859, *p* = 0.014). The bootstrapped indirect effect was ab = 0.081, with a 95% bias-corrected bootstrap confidence interval that excluded zero (95% CI [0.015, 0.174]), indicating that sleep disruption significantly mediated the relationship between smartphone dependency and emotion-regulation difficulties in this corrected analysis (Figure 3). This result supports hypothesis 4. Given the cross-sectional design, however, this finding should be interpreted as an association-level indirect effect rather than evidence of a causal mediating mechanism; the direction and temporal ordering of these relationships cannot be established from these data. An initial analysis conducted on the full N = 167 sample, in which 12 participants with uninterpretable sleep-duration data were scored as 0 rather than excluded, produced a smaller, non-significant indirect effect (ab = 0.060, 95% CI [−0.014, 0.140]); Section 4.4 discusses this discrepancy in detail.

## 4. Discussion

### 4.1. Principal Findings

Although there are many studies that have correlated problematic smartphone use with sleep and affective outcomes in previous studies, the present study is noteworthy for its simultaneous modeling of all three constructs and formal mediation analysis, which yielded three main results. First, smartphone dependency was common: more than one in three respondents (37.7%) screened positive on validated sex-specific SAS-SV cut-offs. Second, all three hypothesized pairwise associations were confirmed smartphone dependency was moderately associated with sleep disruption (r = 0.41) and strongly associated with emotion-regulation difficulties (r = 0.54), and sleep disruption was modestly-to-moderately associated with emotion-regulation difficulties (r = 0.37), and heavier self-reported daily use tracked with higher dependency. Third, once the sleep-duration missing-data issue was corrected (n = 155; Section 4.4), sleep disruption significantly mediated the dependency–emotion-regulation relationship: the bootstrapped indirect effect was ab = 0.081, with a 95% bias-corrected confidence interval that excluded zero (95% CI [0.015, 0.174]), while smartphone dependency retained a strong direct effect that survived adjustment for sleep, age, and sex (β = 0.47). Because the design is cross-sectional, this mediation result is best read as a statistical association-level finding rather than evidence of a causal pathway; it does not establish that sleep disruption causes or definitively explains the dependency–emotion-regulation link, and the direction of these relationships cannot be determined from these data. The significance of these findings is twofold. Clinically, they suggest that both smartphone dependency and the sleep pathway may be relevant targets for intervention, and they quantify a screening-detectable at-risk group of meaningful size. Methodologically, by modeling the three constructs jointly with validated instruments, a formal mediation test, and a corrected approach to missing sleep-duration data, the study addresses a gap left by prior single-construct and screen-time-based designs.

### 4.2. Comparison with Prior Work

The observed prevalence and the dependency–sleep and dependency–emotion-regulation associations are broadly consistent with the problematic-smartphone-use literature, including syntheses linking such use to anxiety, depressive symptoms, and disturbed sleep [3,4,5,11,16,17]. The distinctive contribution here is that, once the sleep-duration missing-data issue was corrected (Section 4.4), the data support the hypothesized sleep-mediated pathway from smartphone dependency to emotion-regulation difficulties, consistent with models that position poor sleep as a plausible conduit from device use to affective difficulty [9,10] and with the neurobiological and meta-analytic evidence for this mechanism discussed in the Introduction [14,15]. Our cross-sectional data show a significant indirect effect through sleep disruption alongside a strong direct effect of dependency on emotion-regulation difficulties that also fits with compensatory and mood-management accounts of problematic use [11,12], though the cross-sectional design does not allow the directionality or temporal mechanism of either pathway to be determined. This reinforces the conceptual distinction between dependency as a behavioral pattern and mere exposure duration [3,4,5], while underscoring that the sleep pathway identified here is a statistical association requiring longitudinal confirmation, not an established causal mechanism, when designing interventions.

### 4.3. Implications for Nursing Practice

Because dependency not hours of use or an intervening sleep pathway carried the strongest and most direct signal. A direct approach to addressing problematic-use patterns that could be considered is led screening and counseling. The approximately one in three youth in this sample who screened in the positive could also be offered early counseling-based intervention, prioritizing education over intervention, in the form of digital-wellness counseling, adaptive coping skill-building, emotion-regulation skill-building, and sleep-hygiene support as an independent wellbeing target and not as the assumed mechanism. Before implementation, the effectiveness, feasibility and clinical benefit of such screening and intervention would have to be proven in prospective studies. Nurses’ established roles in health promotion and behavioral counseling make them well suited to lead such preventive programs [18].

### 4.4. Limitations

Several limitations qualify these findings. The cross-sectional design precludes causal or genuine temporal-mediation inference; even though the corrected mediation analysis (n = 155) found a statistically significant indirect effect, a mediation model estimated from a single time point cannot establish the presumed causal ordering, and this result should be read as an association-level finding rather than evidence of a causal sleep-mediated mechanism. Recruitment was open, voluntary, and snowball-based, which introduces self-selection bias, prevented computation of a view-to-completion rate, and yielded a sample skewed toward female and university-aged respondents, limiting generalizability; cookie/IP deduplication was not enforced, a recognized CHERRIES caveat. The sleep-disruption measure was a five-component PSQI-derived composite with modest internal consistency (α = 0.60); unreliable free-text timing entries required omitting the latency and habitual-efficiency components, which may still limit how precisely this composite captures the validated PSQI construct even after the missing-data correction described below. Relatedly, although the corrected sample (n = 155) exceeded the Cohen-based requirement for the correlational and regression objectives, this remains smaller than the approximately 500 participants originally targeted for the mediation analysis, and a larger sample would provide a more definitive test. Finally, all measures were self-reported and assessed concurrently, raising the possibility of common-method variance. The PSQI and DERS-SF were administered in their original English-language versions rather than a validated regional translation; this reflects the sample’s high English proficiency and English-medium curriculum, but it remains a limitation for generalizing to populations with lower English proficiency, and cross-cultural validation of these instruments in the target population was not established. An earlier version of this analysis, conducted on the full N = 167 sample, scored the 12 participants with uninterpretable sleep-duration data as 0 rather than treating them as missing; this initial analysis produced a smaller, non-significant indirect effect (ab = 0.060, 95% CI [−0.014, 0.140]; Section 3.6). We are grateful to a reviewer who correctly identified that assigning a substantive value of zero to genuinely unknown data was not an appropriate way to handle this missingness, since doing so implicitly assumes the best possible outcome for cases with no actual information. Following this feedback, we corrected the analysis by excluding these 12 cases (complete-case analysis, n = 155) and now report this corrected analysis as primary throughout the manuscript; the initial N = 167 analysis is retained here for transparency. The two analyses differ only in this respect. We note that the corrected result still warrants cautious interpretation: it rests on a smaller sample than originally planned, the sleep composite’s reliability remains modest, and the cross-sectional design cannot establish that sleep disruption causally explains the dependency–emotion-regulation relationship. Separately, exclusion and eligibility criteria including the “other mental health issue” criterion were assessed by self-report only, without clinical verification; because emotion-regulation difficulties are a principal outcome of this study, excluding participants based on a loosely defined, self-identified mental-health criterion introduces a further potential source of selection bias that could have restricted the observed variance in this outcome.

### 4.5. Future Directions

Future research should test the sequential dependency → sleep → emotion-regulation model prospectively, using longitudinal or ecological momentary assessment designs that establish temporal order and can capture within-person dynamics. Larger, probability-based, and more demographically balanced samples ideally across multiple institutions and countries would improve generalizability and provide adequate power for the indirect effect. Incorporating objective measures (e.g., device-logged usage and actigraphy-based sleep) alongside the full PSQI would reduce common-method and self-report bias. Finally, given the strong direct dependency–emotion-regulation link, intervention studies should evaluate whether nurse-delivered digital-wellness and emotion-regulation skills programs reduce dependency and improve affective outcomes, and whether sleep benefits emerge as an independent rather than mediating effect.

## 5. Conclusions

Smartphone dependency is prevalent among young adults and is strongly linked to both disturbed sleep and difficulty regulating emotion. Within the limits of this cross-sectional design, a corrected complete-case analysis (n = 155) found statistically significant evidence that sleep disruption partially mediates the dependency–emotion-regulation relationship, alongside a strong direct effect of dependency itself; an earlier analysis that scored 12 participants with uninterpretable sleep-duration data as zero, rather than excluding them, had found a smaller, non-significant indirect effect. These findings should be interpreted cautiously: they rest on a modestly reliable sleep composite, a sample smaller than originally planned, and a cross-sectional design that cannot establish causal or temporal ordering. Accordingly, nursing interventions could reasonably target smartphone dependency directly, while treating the sleep pathway identified here as a promising but not yet confirmed mechanism warranting further study, alongside continued support for healthy sleep as an independent determinant of young-adult wellbeing. Longitudinal, multi-site research using complete sleep-timing data is needed to confirm the temporal pathways suggested here.

## Figures and Tables

**Table 1:** Sociodemographic characteristics of the analytic sample (N = 167).

Characteristic	n	%
***Sex, n (%)***		
Female	117	70.1
Male	49	29.3
Prefer not to say	1	0.6
***Daily Smartphone Use, n (%)***		
Daily use < 2 h	21	12.6
Daily use 2–5 h	63	37.7
Daily use > 5 h	83	49.7
Age, mean (SD)	21.6 (4.6)	—

n: number of participants; %: percentage of the analytic sample; SD: standard deviation.

**Table 2:** Descriptive statistics and internal consistency of study measures (N = 167).

Measure (Range)	Mean (SD)	Observed Range	Cronbach α
SAS-SV, dependency (10–60)	30.1 (12.5)	10–60	0.94
PSQI-derived sleep disruption (0–15)	5.7 (2.8)	0–15	0.60 ^a^
DERS-SF, ER difficulties (18–90)	48.7 (13.5)	22–78	0.88

^a^ The Cronbach α shown for this row (α = 0.60, n = 155 complete-case) is the internal consistency of the five-component PSQI-derived Sleep Disruption composite actually entered into all analyses (Table 3 and Table 4, Section 3.6), computed on the n = 155 complete-case sample after excluding 12 participants with uninterpretable sleep-duration data. This value reflects the composite’s heterogeneous, differently scaled components. For reference only, α = 0.90 is the reliability of the 9-item PSQI sleep-disturbance subscale (component 5) in isolation; it is not the reliability of the composite score used here and was reported in error in the previously submitted version. SAS-SV: Smartphone Addiction Scale–Short Version; PSQI: Pittsburgh Sleep Quality Index; DERS-SF: Difficulties in Emotion Regulation Scale–Short Form; ER: emotion regulation.

**Table 3:** Pearson correlations among study variables (*n* = 155).

Variable	1	2	3
1. Smartphone dependency	—		
2. Sleep disruption	0.41 ***	—	
3. ER difficulties	0.54 ***	0.37 ***	—

*** *p* < 0.001. Correlations computed on n = 155 (excluding 12 participants with uninterpretable sleep-duration data); see Section 3.6 and Section 4.4 for comparison with the initial N = 167 analysis. ER: emotion regulation.

**Table 4:** Multiple linear regression predicting emotion-regulation difficulties (DERS-SF) (*n =* 155).

Predictor	B (SE)	β	*p* Value
Smartphone dependency	0.52 (0.08)	0.47	<0.001
Sleep disruption	0.86 (0.35)	0.18	0.01
Age	−0.14 (0.19)	−0.05	0.48
Female (vs. male)	−1.38 (1.95)	−0.05	0.48

B: unstandardized coefficient; β: standardized coefficient (from the same standardized four-predictor model shown in this table). Model (n = 155): R^2^ = 0.33, F_4,150_ = 18.02, *p* < 0.001. Regression diagnostics (n = 155): all VIF ≤ 1.22 (no multicollinearity); Durbin–Watson = 1.90; Cook’s D identified 5 observations above the 4/n threshold, none exceeding 1.0; residuals did not show significant non-normality (Jarque–Bera *p* = 0.18). VIF: variance inflation factor.

## Data Availability

De-identified datasets remain available from the corresponding author upon reasonable request due to participant confidentiality.

## Supplementary Materials

The following supporting information can be downloaded at https://www.mdpi.com/article/10.3390/healthcare14182982/s1, File S1: STROBE Statement—Checklist of items that should be included in reports of cross-sectional studies.

## Abbreviations

DERS-SF: Difficulties in Emotion Regulation Scale–Short Form; PSQI: Pittsburgh Sleep Quality Index; SAS-SV: Smartphone Addiction Scale–Short Version; SD: standard deviation; SEM: standard error of the mean; CHERRIES: Checklist for Reporting Results of Internet E-Surveys.
