# Supplementary material for: Beyond Screen Time: Smartphone Dependency, Sleep Disruption, and Emotional Regulation Among Young Adults—A Cross-Sectional Web-Based Survey
Source: Healthcare (Basel). 2026 Sep 12;14(18):2982. doi: 10.3390/healthcare14182982 (PMC13606723; doi:10.3390/healthcare14182982)
Supplement: Supplementary file 1 [file healthcare-14-02982-s001.zip › healthcare-4517881-supplementary.pdf]

Supplementary File 1

STROBE Statement—Checklist of items that should be included in reports of cross-sectional studies

Manuscript: "Beyond Screen Time: Smartphone Dependency, Sleep Disruption, and Emotional Regulation Among Young Adults—A Cross-Sectional Web-Based Survey."

Page numbers refer to the paginated main-text file (15 pages). Reconfirm them against the final typeset manuscript if the text changes before submission.

Item	Topic	Recommendation	Page(s)
	Title and abstract		
1	**Title and abstract**	(a) Indicate the study design with a commonly used term in the title or the abstract. (b) Provide in the abstract an informative and balanced summary of what was done and what was found.<br>*Addressed in the Title ("A Cross-Sectional Web-Based Survey") and the structured Abstract (Background, Objective, Methods, Results, Conclusions).*	1–2
	Introduction		
2	**Background/rationale**	Explain the scientific background and rationale for the investigation being reported.<br>*Addressed in Section 1.1 (Background).*	2–3
3	**Objectives**	State specific objectives, including any prespecified hypotheses.<br>*Addressed in Section 1.2 (Objectives and Hypotheses): six objectives and prespecified hypotheses H1–H4.*	3
	Methods		
4	**Study design**	Present key elements of study design early in the paper.<br>*Addressed in Section 2.1 (Study Design): cross-sectional, descriptive-analytical web survey reported per CHERRIES and STROBE.*	3
5	**Setting**	Describe the setting, locations, and relevant dates, including periods of recruitment, exposure, follow-up, and data collection.<br>*Addressed in Section 2.2 (Recruitment, Setting, and Researcher Independence): anonymous web survey disseminated via social media across regions. (IRB approval KFU-REC-2026-AUG– ETHICS4413 on p. 13; an explicit data-collection window is not stated and could be added.)*	4
6	**Participants**	(a) Give the eligibility criteria, and the sources and methods of selection of participants.<br>*Addressed in Sections 2.2 and 2.3 (Participants and Eligibility): inclusion (≥18 years, smartphone owner), exclusion criteria, and snowball/social-media selection.*	4
7	**Variables**	Clearly define all outcomes, exposures, predictors, potential confounders, and effect modifiers. Give diagnostic criteria, if applicable.<br>*Addressed in Section 2.5 (outcome: DERS-SF emotion-regulation difficulties; exposure: SAS-SV dependency; mediator: PSQI-derived sleep disruption) and Section 2.7 (covariates age and sex).*	5–6
8	**Data sources/measurement**	For each variable of interest, give sources of data and details of methods of assessment (measurement). Describe comparability of assessment methods if there is more than one group.<br>*Addressed in Section 2.5 (Measures and Data Collection Procedure): four-section instrument, scale scoring, and Python data cleaning.*	5
9	**Bias**	Describe any efforts to address potential sources of bias.<br>*Addressed in Section 2.2 (investigator independence and anonymity to reduce interviewer and social-desirability bias; embedded eligibility screening) and Section 4.4 (self-selection, coverage, and common-method variance).*	4, 12
10	**Study size**	Explain how the study size was arrived at.<br>*Addressed in Section 2.4 (Sample Size and Statistical Power): a priori power analysis and achieved N=167 with precision estimates.*	4
11	**Quantitative variables**	Explain how quantitative variables were handled in the analyses. If applicable, describe which groupings were chosen and why.<br>*Addressed in Section 2.5 (composite scoring of SAS-SV, PSQI-derived score, and DERS-SF; daily-use bands) and Section 2.7 (Statistical Analysis).*	5–6
12	**Statistical methods**	(a) Describe all statistical methods, including those used to control for confounding. (b) Describe any methods used to examine subgroups and interactions. (c) Explain how missing data were addressed. (d) If applicable,	5–7

Item	Topic	Recommendation	Page(s)
		describe analytical methods taking account of sampling strategy. (e) Describe any sensitivity analyses. *Addressed in Section 2.7 (Statistical Analysis): correlations, t tests, ANOVA, covariate-adjusted regression, bootstrapped mediation, Bonferroni/FDR corrections; missing-data handling (≥80% completion rule) in Section 2.5; power/precision in Section 2.4.*	
	Results		
**13**	**Participants**	(a) Report numbers of individuals at each stage of study. (b) Give reasons for non-participation at each stage. (c) Consider use of a flow diagram. *Addressed in Sections 2.3 and 3.1: 172 responses collected → 3 declined, 2 ineligible, cases with missing analytic data removed → 167 analysed. (No participant flow diagram is included; item 13c is optional.)*	4, 7
**14**	**Descriptive data**	(a) Give characteristics of study participants and information on exposures and potential confounders. (b) Indicate number of participants with missing data for each variable of interest. *Addressed in Section 3.1 and Table 1; missing-data counts noted in the Table 2 footnote (n=155 for the sleep composite) and Section 4.4 (12 cases missing sleep-duration).*	7, 8, 12
**15**	**Outcome data**	Report numbers of outcome events or summary measures. *Addressed in Section 3.2 (Descriptive Statistics, Reliability, and Prevalence) and Table 2: composite means (SD) and 37.7% screen-positive prevalence.*	7–8
**16**	**Main results**	(a) Give unadjusted estimates and, if applicable, confounder-adjusted estimates and their precision (e.g., 95% confidence interval). (b) Report category boundaries when continuous variables were categorized. (c) If relevant, consider translating estimates of relative risk into absolute risk. *Addressed in Sections 3.3 (correlations with Fisher-z 95% CIs), 3.4 (group differences), 3.5 (age/sex-adjusted regression), and 3.6 (bootstrapped mediation with 95% CI); Tables 3 and 4; daily-use category boundaries defined in Sections 2.5 and 3.4.*	8–11
**17**	**Other analyses**	Report other analyses done—e.g., analyses of subgroups and interactions, and sensitivity analyses. *Addressed in Section 3.4 (sex and daily-use subgroups), Section 3.1 (restricted-sample sensitivity), Section 2.7 (Bonferroni/FDR), and Section 2.5 (sleep-scoring sensitivity).*	7, 8, 9
	Discussion		
**18**	**Key results**	Summarise key results with reference to study objectives. *Addressed in Section 4.1 (Principal Findings).*	11
**19**	**Limitations**	Discuss limitations of the study, taking into account sources of potential bias or imprecision. Discuss both direction and magnitude of any potential bias. *Addressed in Section 4.4 (Limitations).*	12
**20**	**Interpretation**	Give a cautious overall interpretation of results considering objectives, limitations, multiplicity of analyses, results from similar studies, and other relevant evidence. *Addressed in Sections 4.2 (Comparison with Prior Work), 4.3 (Implications for Nursing Practice), 4.5 (Future Directions), and 5 (Conclusions).*	11–13
**21**	**Generalisability**	Discuss the generalisability (external validity) of the study results. *Addressed in Section 4.4 (Limitations): female- and university-skewed, self-selected sample and English-language instruments.*	12
	Other information		
**22**	**Funding**	Give the source of funding and the role of the funders for the present study and, if applicable, for the original study on which the present article is based. *Addressed in the Funding statement (Deanship of Scientific Research, King Faisal University; Grant No. KFU264287).*	13

An Explanation and Elaboration article discusses each checklist item and gives methodological background and published examples of transparent reporting. The STROBE checklist is best used in conjunction with this article, available on the STROBE website (https://www.strobe-statement.org).
